# Prakriti (constitutional typology) in Ayurveda: a critical review of Prakriti assessment tools and their scientific validity

**DOI:** 10.3389/fmed.2025.1656249

**Published:** 2025-11-06

**Authors:** Archana Venkatesh, Lina Johansson, Prabu Vignesh Sivanandan, Shiva Pratap Gopakumar, Karthik Sankaranarayanan, Christian S. Kessler, Shraddha Ravani, Rammanohar Puthiyedath

**Affiliations:** 1 Amrita School of Ayurveda, Amrita Vishwa Vidyapeetham, Kollam, India; 2 Imperial College Healthcare NHS Trust, London, United Kingdom; 3 Department of Diabetes, Endocrinology and Metabolism, Imperial College London, London, United Kingdom; 4 Netcracker Technology, Bengaluru, India; 5 Amrita School of Sustainable Futures, Amrita Vishwa Vidyapeetham, Kollam, India; 6 Faculty of Business and Information Technology, Ontario Tech University, Ontario, ON, Canada; 7 Charité Competence Center for Traditional and Integrative Medicine (CCCTIM), Charité - Universitätsmedizin Berlin, Corporate Member of Freie Universität Berlin and Humboldt Universität zu Berlin, Berlin, Germany; 8 Department of Internal Medicine and Nature-Based Therapies, Immanuel Hospital Berlin, Berlin, Germany; 9 Department of Physiology and Health Maharishi International University, Fairfield, IA, United States

**Keywords:** Prakriti, Ayurveda, body constitutional typology, personalized health care, technology integration, measurable correlates

## Abstract

**Background:**

Prakriti or constitutional typology is the foundation of personalized health care in Ayurveda. Traditionally, Ayurvedic clinicians have assessed Prakriti in a primarily experience-based and often subjective manner. However, in the past few decades attempts to develop objective tools have been made by researchers from multidisciplinary domains. This review aimed to identify existing Ayurvedic Prakriti assessment tools and evaluate their scientific rigor.

**Methods:**

Aligned with the SANRA framework, our narrative review incorporated systematic elements. A Boolean search in PubMed, Scopus, and Cochrane in November 2024 using (“Prakriti”) AND (“Ayurveda” OR “Ayurvedic”) yielded 635 articles, together with 12 additional articles from citations search. Ninety four studies met the inclusion criteria. Prakriti assessment tools were quantified and evaluated using Scale Development and Validation Framework by Boateng et al., alongside custom set of study quality indicators to assess their methodological rigor.

**Results:**

Between 1987 and 2024, 64 unique Prakriti assessment tools (PATs) were identified, each using one or more methods to perform data collection and decision-making tasks. Variations in the selection and application of these methods resulted in the development of diverse methodological frameworks for Prakriti assessment. Of the 64 PATs identified, only 20 PATs underwent any form of validation and among them, just two PATs, the CCRAS-PAS software and ACPI scale met seven of the nine recommended criteria. Most tools lacked dimensionality testing, test–retest reliability, contextual validity and were not tested across diverse populations, indicating a high risk of developer-bias. Additionally, 32 categories of measurable correlates to Prakriti have been studied across 94 studies, but only five of them were studied using validated tools.

**Conclusion:**

Much progress has been made in developing methodology and integrating technology for creating Prakriti assessment tools along with attempts to identifying measurable correlates to Prakriti that could potentially serve as Prakriti biomarkers. Currently no tool fully meets the evaluation criteria of the Scale Development and Validation framework, except CCRAS-PAS and ACPI that show partial readiness and can be refined. Further work is needed to establish Prakriti as a clinically validated measurable construct and to integrate Ayurveda into the domain of personalized health care.

## 1 Introduction

Ayurveda translates to “Knowledge of life.” It is the traditional knowledge system from India that offers a comprehensive understanding of life, health, longevity, along with the therapeutic aspects. The earliest texts of Ayurveda had attained a high level of systematization by 500 BCE, though the tradition of Ayurveda dates back much earlier (1). The use of Ayurveda is still very prominent in India (2), with a growing global academic interest. Academic training programs in Ayurveda are now offered by dedicated Ayurveda institutions as well as established universities across Europe, United States, Canada, Australia (3). A recent study showed that Ayurveda was preferred for its “natural approach” and “fewer side effects” by patients in the Organization for Economic Cooperation and Development (OECD) countries for managing non-communicable diseases (NCDs) (4).

Personalized treatment is an integral part of Ayurvedic clinical practice (5) which involves implementing multiple therapeutic approaches to treat different people with the same diagnosis. With its culturally-sensitive and holistic approach, Ayurveda also offers better affordability and accessibility (6).

Recent advances in personalized medicine has made it possible to predict disease susceptibility and make early detection through genetic, genomic, and other individual-level profiling (7). It allows physicians to personalize preventive, promotive, and therapeutic strategies using approaches like pharmacogenetics (8) with promising applications in family medicine and primary care. However, personalized medical care remains inaccessible for much of the global population (8). Given the rising burden of diseases (9) and the urgent need for affordable and, accessible primary care, traditional systems like Ayurveda could play a pivotal role.

The Ayurvedic concept of Prakriti is at the core of understanding health, disease, and therapeutic intervention for personalized treatment across the clinical care continuum. While all individuals are composed of the same elemental constituents, Ayurveda emphasizes variation in their configuration, expressed through Deha Prakriti (physical constitution) (10–13) and Manasika Prakriti (mental constitution) (14). This aligns with modern scientific understanding that, despite shared biomolecular and cellular components, individuals differ in genetic, epigenetic, metabolic, and other profiles.

Traditionally, Prakriti was assessed through clinical methods such as Trividha Pariksha (15), using observation, palpation and interrogation; Astavidha Pariksha (16) using pulse, urine, feces, tongue, sound, touch, eyes, physique and other methods. While these methods are foundational, they were inherently subjective, limiting their reproducibility and broader clinical applicability.

Early assessments were typically conducted by a single physician, relying heavily on their individual judgment. This approach lacked clarity regarding the domains and data points that informed the Physician’s final Prakriti classification, making it highly subjective. Over time, efforts to address this subjectivity have led to the adoption of more objective approaches, including the use of pulse assessment, a questionnaire or digital interfaces that standardize data entry, and more advanced computational techniques such as Machine Learning and Computer Vision. Collectively, these developments show a progressive shift from highly subjective to increasingly structured and objective methods, laying the groundwork for the diverse Prakriti assessment tools that exist today.

### 1.1 Theoretical construct of Prakriti as a measurable clinical parameter

Prakriti in Ayurveda involves constitutional phenotyping, defined as an individual’s inherent psychophysical constitution established at the time of conception (12, 13). Prakriti is considered to remain unchanged (13, 17, 133) across the lifespan and influences an individual’s physical features, physiological responses, psychological tendencies, behavioral patterns, disease susceptibility, and response to medical interventions. The classical texts of Ayurveda have provided 200–250 characteristics. For example, body build, frequency of hunger, skin complexion, sleep patterns, voice characteristics, tolerance to temperature, taste preference, mental temperament, encompassing physical, physiological, psychological and behavioral traits (18). Therefore, Prakriti represents a multidimensional construct comprising both observable traits and latent dimensions that require systematic exploration. Much like the constructs of psychology and personality used in Behavioral medicine, Prakriti also requires measurement through structured instruments.

The physical constitution reflects a unique configuration of the three doshas (Tridosha) of Vata, Pitta, and Kapha (134) resulting in seven primary types. The mental constitution represents the configuration of the three mental attributes (Trigunas) i.e., Sattva, Rajas, and Tamas that are classified into 16 types (14). While both the physical and mental constitution influence psychological traits, the mental constitution also influences moral disposition and spiritual inclinations. Ayurveda conceptualizes health as a dynamic balance between the physical and mental domains, and the root cause of disease is due to the disturbances in the doshas, caused by various factors including the mind. Therefore, though physical and mental constitution are described as conceptually distinct in the classical texts, they exert a combined influence on the mind of the individual. Among the two, the physical constitution has been more widely studied due to its measurable physical attributes.

The classification of physical constitution helps in assessing disease predisposition, customizing diet and lifestyle recommendations, and guiding therapeutic choices, highlighting its alignment with personalized medicine. Its conceptual foundation in Tridosha theory, pool of observable traits, its independence from transient states like disease (Vikriti) allows a trait-based assessment. The stable nature and multidomain expression of an individual’s physical constitution meet key criteria for clinical scale development, with the potential to be translated as a clinically measurable parameter.

However, difficulties in relation to Prakriti tool development exist such as absence of standardized trait definitions, contextual variability (age, geography, season) (10, 132), and the confounding influence of Vikriti (disease), which can obscure baseline traits. For example, the influence of hypothyroidism on the voice characteristics (19).

Over the past three decades, efforts have been made to create a scoring system based on the available 200–250 characteristic traits to make Prakriti a measurable parameter. For example, the physical traits like height and build of an individual have been measured using anthropometric methods such as body mass index (20, 21), image analysis for hair characteristics and facial features (18, 22); physiological functions like bowel health have been measured using microbiome analysis (23–27); psychological traits have been measured using validated scales such as the Big Five Inventory (BFI) to assess personality (28, 29). Additionally, researchers have also attempted to correlate Prakriti classifications with various genetic and genomic factors (30–33), which has led to the advancement of research related to Prakriti assessment.

In this background, several Prakriti assessment tools (PATs) have been developed using various methods. A PAT has two core functions to be performed, data collection and decision-making. Different methods have been used to perform these tasks in a PAT that brings out the variability between the PATs. As highlighted by Bhalerao and Patwardhan, the methodologies and tools used for Prakriti assessment have several issues such as conceptual ambiguities and lack of methodological clarity (34). Recognizing Prakriti as a measurable clinical parameter allows its evaluation through frameworks like that of Boateng et al. (35), which emphasize clear domain definition and identification, structured item analysis and reduction for a rigorous scale development and empirical validation. Positioning Prakriti within such a framework supports its transition from classical diagnostic insight to a reliable, evidence-based tool for personalized care.

Therefore, our study aims to quantify the different tools used for Prakriti assessment and evaluate the validity of these tools. To our knowledge, this is the first review to systematically evaluate PATs using a tool development and validation framework. This represents a first step toward bridging the gap between Prakriti as a foundational Ayurvedic concept and its translation to a clinically measurable parameter.

## 2 Methodology

This is a narrative review, adhering to the SANRA framework—a scale for the quality assessment of narrative review articles (36) while using elements of the PRISMA flow diagram designed for systematic reviews (37) to present the study selection process. The methodology evolved through multiple stages: formulating review questions, conducting the literature search, selecting articles for the review, charting and data synthesis, and data analysis.

We framed the narrative synthesis based on two questions:

1. What are the different tools available for Prakriti assessment?
2. Do the currently available Prakriti assessment tools adequately meet established standards for tool development and validation?

### 2.1 Inclusion criteria

• Types of research articles included:

o Studies focusing on the development, validation, or comparison of assessment tools for the assessment of Prakriti.
o Quasi-experimental or experimental clinical studies, scoping review, narrative review, and systematic reviews that involve the assessment of Prakriti were also included.
o Original research articles published in journals or conference proceedings.
o Abstract-only articles providing relevant details on Prakriti assessment methods and mentioning specific assessment tools.

• Language:

o Only studies published in English were included in the review.

• Population:

o Studies conducted on human participants of any age, gender, or ethnicity where Prakriti assessment was performed were eligible.

• Quality criteria for article and journal selection:

o Compliance with the article reporting guidelines was the basis for inclusion of articles, irrespective of whether the article was published in a non-indexed or indexed journal.

### 2.2 Exclusion criteria

• Relevance:

o Studies that lacked identifiable Prakriti assessment tools or methods were excluded.
o Articles with insufficient data to contribute to the data synthesis for the review were excluded.

• Type of research articles:

o Reviews, opinion pieces, commentaries, and book chapters that did not look at Prakriti assessment and did not contribute to the data synthesis of the review were excluded.

• Type of study subjects:

o Research conducted on animal models or *in vitro* studies.

• Quality criteria:

o Articles from gray journals, i.e., journals not indexed in reputed databases such as Scopus, PubMed, or Cochrane Library; journals not published by respectable publishers such as Springer, Nature, Elsevier; journals that made unverifiable claims about its impact factor or editorial board were excluded.
o Articles or thesis that have not undergone rigorous peer-review process and not published in the indexed journals such as Scopus, PubMed, or Cochrane Library were also excluded.

### 2.3 Search strategy

The search was conducted in November 2024. The search strategy aimed to identify published studies exploring the use of tools or methods for Prakriti assessment. Various keyword combinations were tested across three databases—Scopus, PubMed, and Cochrane Library. The study used Boolean operators to refine searches, with no time restriction and the final combination being:

(“Prakriti”) AND (“Ayurveda” OR “Ayurvedic”)

### 2.4 Study selection process

**Figure 1:**
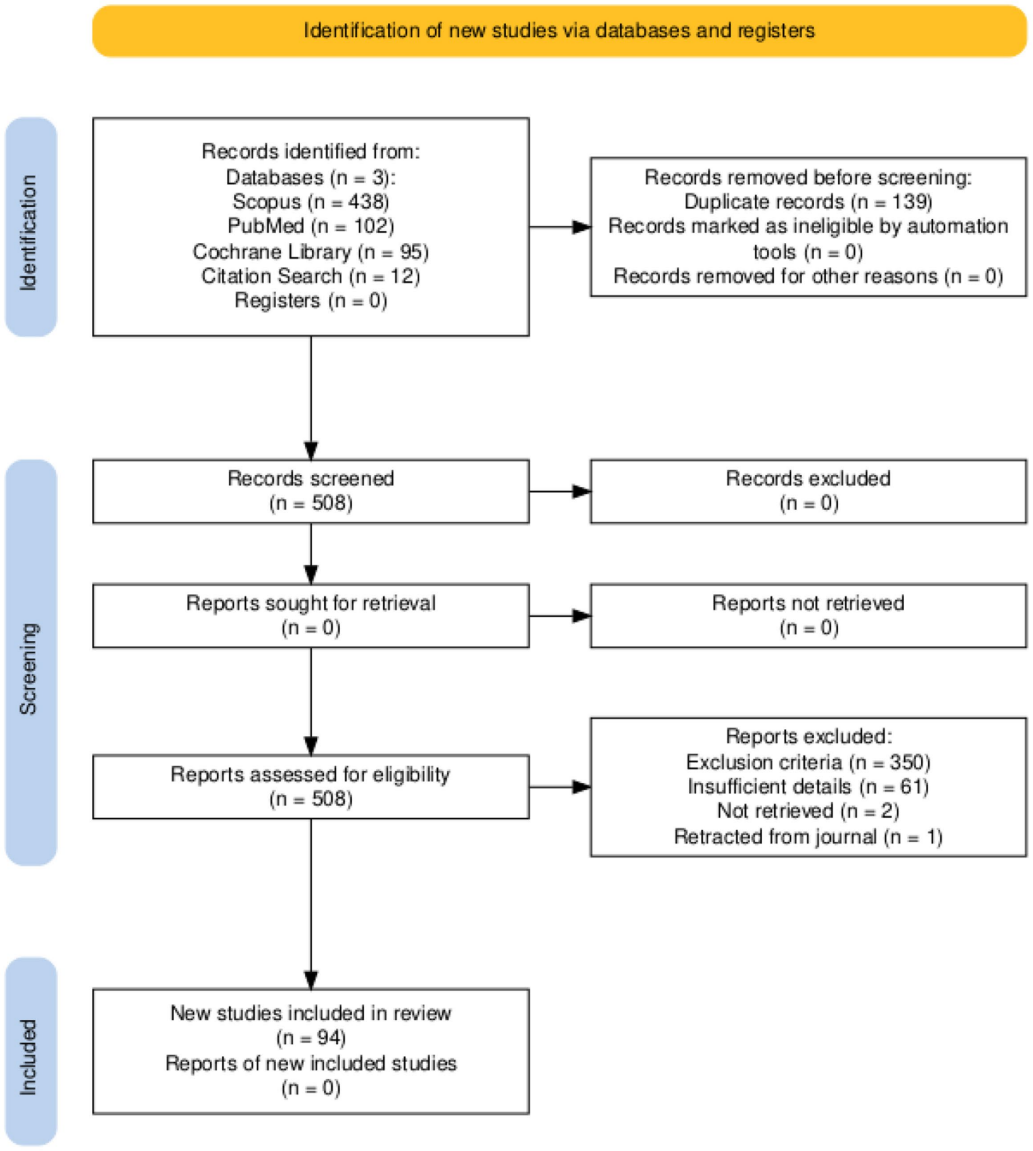
PRISMA flow diagram.

All identified citations were exported as CSV files, with or without abstracts. A total of 635 records were retrieved through database searches, and 12 papers were added to the pool based on citation search. One hundred and thirty-nine duplicates were identified using pivot tables in Microsoft Excel, which were excluded from the analysis. Of the remaining 508 records screened for eligibility, 350 were excluded based on the exclusion criteria, 61 due to insufficient details, 2 could not be retrieved, and 1 was a retracted study. This brought the total number of included reports to 94 published between 1987 and 2024. We have presented the results using a PRISMA flow diagram (see Figure 1).

### 2.5 Charting, data synthesis and analysis

A single reviewer reviewed each selected paper. We addressed the single reviewer bias to some extent by using a holistic scoring rubric, to make the data extraction objective (see Supplementary Appendix 1). Any doubts or concerns raised by the reviewer were clarified by the co-authors of this paper.

The data from 94 papers were charted and synthesized to answer the two study objectives. To address the first review question, we looked for identifiable Prakriti assessment tools (PATs). An identifiable tool would have a unique name as given by a researcher or research group like “AyuSoft software” (38) or “Mysore Tridosha Scale” (39). In the absence of a unique name, a name was coined by our study team, based on the method employed by the tool, followed by the researcher’s name and the year it was developed or name of the research group. For example, as part of the CSIR consortium project, a software tool was developed but not formally named; therefore, the term “CSIR software” (33) was coined for this PAT.

For the purpose of this review, each PAT was systematically analyzed with respect to the methods used for (i) data collection and (ii) decision-making. This enabled us to identify the range of approaches adopted for gathering information and the mechanisms through which final Prakriti classifications were derived. Particular attention was given to whether the final classification depended on the physician’s interpretation of collected data or whether it was determined autonomously. This distinction allowed us to examine variations in the degree of subjectivity versus automation across PATs.

**Figure 2:**
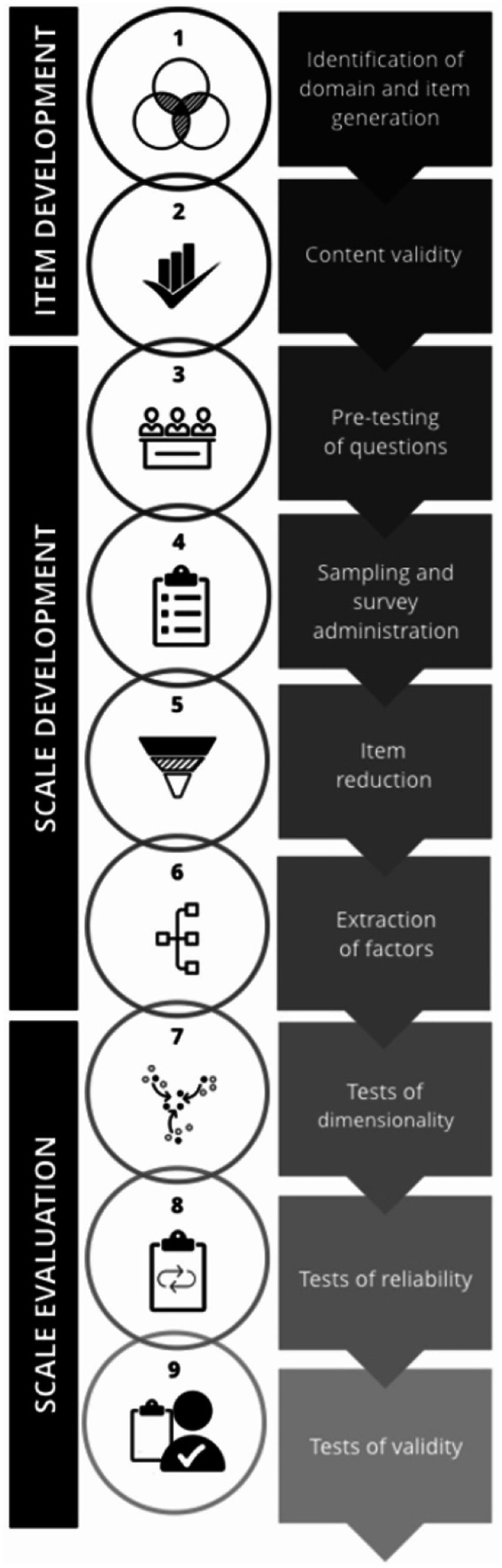
An overview of the three phases and nine steps of scale development and validation. Reprinted from Boateng et al. (35), licensed under CC BY-NC 4.0.

Addressing the second review question which to establish the validity and reliability of Prakriti assessment tools, was particularly challenging due to the lack of an established standard for evaluating Prakriti assessment methods and tools. To address this methodological gap, we conducted a targeted literature search to identify the most appropriate framework for evaluation. The search yielded six relevant sources, comprising one book (40) and five journal articles (35, 41–44) focused on scale development, validity, and reliability testing. Among these, the “Scale Development and Validation Framework” by Boateng et al. (35), was selected for this study. We have used the original figure developed by the authors, which provides an overview of the three phases, and nine steps of scale development and validation has been provided (see Figure 2). This framework is frequently cited and emphasizes best practices for developing and validating scales in health, social, and behavioral research. It offers a structured evaluative approach with specific estimates suitable for critically appraising tools designed to measure complex phenomena or constructs like Prakriti.

We evaluated the tools and their adherence to the prescribed methodological steps in the framework using the rubric. Each PAT was qualitatively evaluated against the nine steps of the Boateng framework using a scoring rubric (Supplementary Appendix 1); fulfillment of at least one specific analysis or procedure per step (e.g., expert panel review for content validity, EFA/CFA for dimensionality) was considered sufficient to score that step as achieved. If no evidence was reported for a given step, the tool received a score of 0 for that step, whereas tools that provided partial evidence (e.g., only expert panel review without CVR/CVI for content validity) received 1 point, ensuring consistent scoring while accommodating methodological variation across studies.

This approach enabled us to perform a structured, comparative analysis of methodological rigor across tools, providing insight into their robustness. However, it must be noted that the scoring was done purely based on what has been reported in the available literature.

Given the inherent complexity of assessing Prakriti, which is influenced by diverse factors, it was recognized that evaluating the study quality, particularly in terms of sample size was critical for appraising the robustness of assessment tools used in the study. Special emphasis was placed on five key factors considered to influence Prakriti: ethnicity (Jati), family lineage (Kula), age (Vaya), geographic location (Desha), and season (Kala) (10). We also examined the range of study designs employed across the 94 included studies. Collectively, the sample size, the five influencing factors and the type of study design were called as “Study Quality Indicators” for the purpose of this evaluation.

In addition to these, we categorized the measurable correlates that have been studied in relation to Prakriti, including genomic, genetic, physiological, biochemical parameters etc., using various PATs.

We conducted a descriptive analysis of the synthesized data using Pivot Tables in Microsoft Excel and the results have been presented in the following section.

## 3 Results

We have presented the results through five sub-sections. The first sub-section lists all the identified PATs and classifies them based on technology integration. The second sub-section presents the evaluation of the tool development and validation based on the Boateng et al., framework. The third sub-section shows the analysis using study quality indicators and the fourth sub-section examines the extent to which these tools have been used to investigate measurable correlates to Prakriti.

### 3.1 Prakriti assessment tools across 94 studies

We identified and cataloged 64 unique Prakriti assessment tools (PATs) (see Table 1).

**Table 1:** Year-wise list of unique PATs.

No.	Year	Tool name
1	1987	Q (88)
2	2005	CA (30)
3	2008	CSIR software (33)
4	2010	Q (53)
5	2011	Mysore Tridosha scale software (39)
6	2011	Q (89)
7	2011	SAQ (90)
8	2010	AyuSoft software (38)
9	2012	CCRAS Q (91)
10	2012	CPA and CA of hand (55)
11	2012	Prototype Prakriti Analysis Tool (PPAT) (92)
12	2012	SAQ (93)
13	2012	Survey Q (94)
14	2012	TNMC Prakriti 2004 Q (95)
15	2013	SAQ (96)
16	2013	SAQ-ABC (97)
17	2013	TSSC SAQ-Tridosha State Scale for Children (47)
18	2014	ACPI scale- Ayurveda Child Personality Inventory (46)
19	2014	Sushruta Prakriti Inventory (SPI-Q & SPI-C) (50)
20	2015	SRQ (QDAV-R) (29)
21	2015	Survey Q (98)
22	2016	ML & Q (99)
23	2017	CCRAS-PAS software (45, 100)
24	2017	ML-Gathered Data (101)
25	2017	SRQ (102)
26	2018	Q (103)
27	2018	Q (104)
28	2018	Q (54)
29	2018	Q (105)
30	2019	Mathew IAS rating Scale (48)
31	2019	ML & CA (106)
32	2019	Q (107)
33	2019	Q (23)
34	2019	Q (52)
35	2019	Q (108)
36	2019	SAQ (109)
37	2019	SAQ Prakruti Dosha Mind Body Quiz and Vikruti Subdosha Questionnaire (28)
38	2020	ML & Q (49)
39	2020	Q (110)
40	2020	RGB analysis (111)
41	2020	SAQ (112)
42	2020	ML & SAQ (113)
43	2021	Portable Radial Pulse [VPK] Signal Acquisition and Recording System (114)
44	2021	Prakriti Q modified for T2DM (115)
45	2021	PRAS-IPA software (22)
46	2021	SAQ (116)
47	2021	SAQ (117)
48	2021	VIYETT Ayurvedic-Constitution Q (20)
49	2022	CPA & Q (118)
50	2022	Q (119)
51	2022	SAQ (51)
52	2022	SAQ (120)
53	2023	CV, ML & CPA (121)
54	2023	ML-Gathered Data (122)
55	2023	Prakriti Assessment Tool (PAT) (123)
56	2023	Q (124)
57	2023	ML & Q (125)
58	2023	SAQ (126)
59	2024	CPA method (127)
60	2024	CV, ML & CA (18)
61	2024	ML & CA (128)
62	2024	ML-Smart device data (129)
63	2024	Q (130)
64	2024	CPA & SRQ (131)

#### 3.1.1 Diverse methodological frameworks adopted by PATs

**Figure 3:**
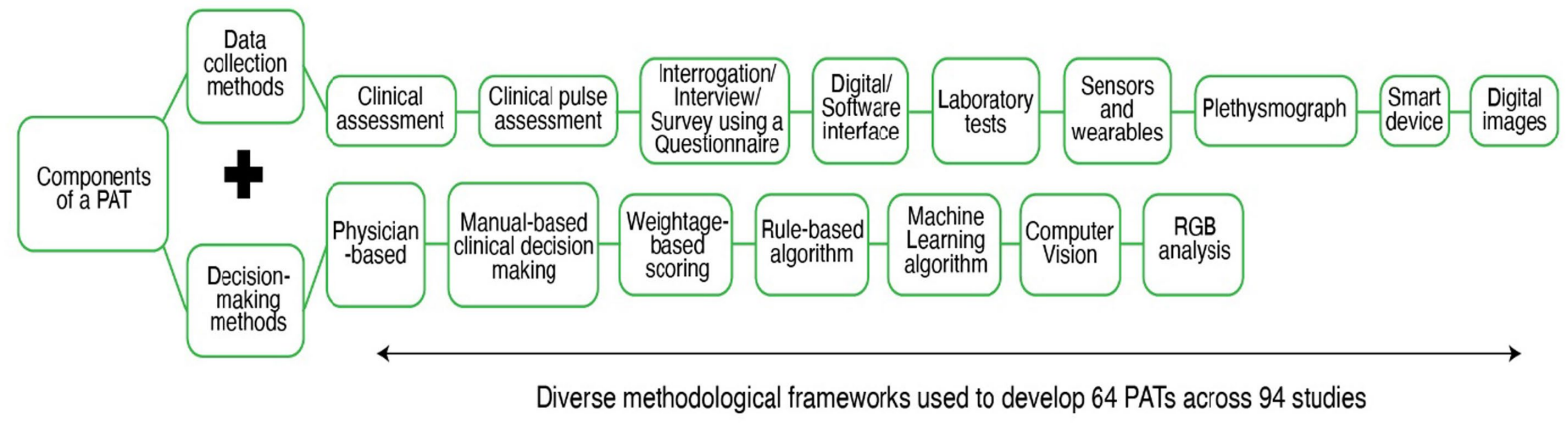
Data collection and Decision-making methods used across 64 PATs.

Initially, we wanted to identify and quantify the tools used for Prakriti assessment, but we found that every PAT varied in terms of the methods they used for data collection, and decision-making, resulting in diverse methodological frameworks being used for Prakrit assessment (see Figure 3). The heterogeneity between tools was evident not only at the level of frameworks but also within each functional domain. In data collection, tools differed in the domains assessed (e.g., physical, physiological, psychological traits) and in the specific data points used for Prakriti evaluation. In decision-making, the degree of subjectivity versus objectivity varied according to the methods employed. For instance, some studies relied on clinical assessments performed by multiple physicians, while others used algorithmic approaches that reduced or eliminated physician involvement.

#### 3.1.2 Trends related to technology integration of PATs

**Figure 4:**
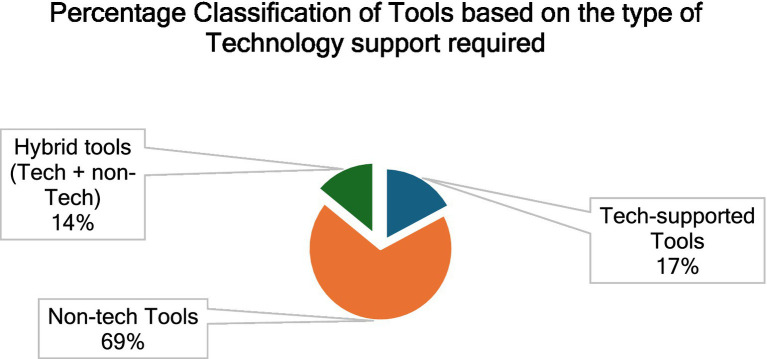
Percentage classification of the tools based on the type of technological support required.

Out of the 64 PATs, there were 41 Questionnaires and 4 software. Other PATs involved the use of different methodological frameworks with varying degrees of Physician involvement and technology use. Figure 4 presents the percentage classification of the tools based on the nature of technological support they require. It must also be noted that, while the tech-supported tools and hybrid tools cannot be used without the support of a hardware (computers, sensors, plethysmograph etc.) and/or software (web-based or offline applications, MATLAB etc.), the non-tech tools could be used manually to perform the Prakriti assessment. Out of 64 tools, we found 44 non-tech tools; 11 tech-supported tools and 9 hybrid tools (tech + non-tech). We also found that though newer techniques like Machine Learning (ML) and Computer Vision (CV) have been used for Prakriti assessment, they still exist as frameworks and have not been developed into usable tools.

Due to the variability in the involvement of Physician(s) and technology use, the PATs either served as a Decision support system (DSS) or an autonomous Decision-making system (DMS). While most PATs operated as autonomous DMS, only one PAT, AyuSoft, functioned as a DSS. The AyuSoft software enabled structured data collection, and the decision-making depended on physician-defined weightages.

### 3.2 Tool evaluation based on the scale development and validation framework

**Figure 5:**
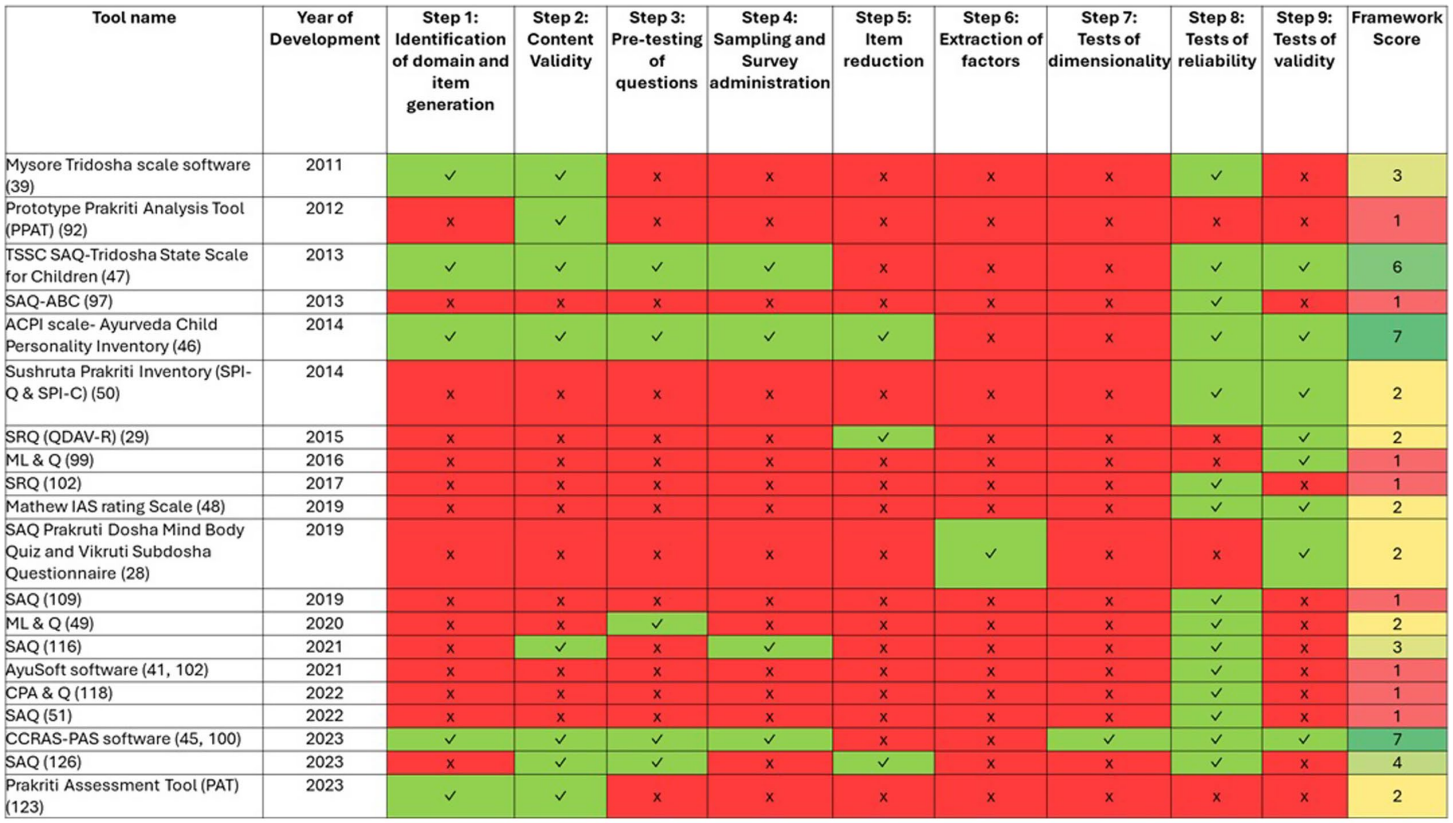
Validation of Prakriti Assessment Tools (PATs) against scale development and evaluation criteria (1987–2024). Each row represents a PAT, and each column represents one of the nine evaluation criteria. Green cells (✓) indicate the criterion was fulfilled, red cells (✗) indicate it was not fulfilled. The numbers in the rightmost column indicate the total count of criteria fulfilled for each tool, where shades of green color indicate PATs scoring >4; shades of yellow indicate PATs scoring 2–4 and red indicate PATs scoring <2.

We evaluated all the identified PATs using a holistic scoring rubric (Supplementary Appendix 1) based on the identified Framework by Boateng et al. (35), and found that out of 64, only 20 PATs have undergone some form of validity and reliability testing using standard methods. The result of this analysis is given in Figure 5.

We found that no tool looked at all 9 steps spanning the three phases listed in the framework. Two tools, the CCRAS-PAS software (45) and the Ayurveda Child Personality Inventory (ACPI scale) (46) fulfilled seven out of nine steps defined in the evaluation framework and one tool, the TSSC-SAQ (47) fulfilled 6 of the 9 steps.

Tools scoring below 5 of 9 often skipped early phases of the item and scale development and relied on inadequate validity and reliability testing. Many tools scoring ≤3 omitted the first two phases of item and scale development. Among the 20 PATs, most focused on assessing the physical constitution, only one tool- the Mathew IAS rating Scale (48) assessed mental constitution.

Nineteen out of twenty tools were tested by their developers, except the widely used AyuSoft software, which was externally tested for internal consistency along with Intra-class correlation coefficient (ICC) for inter-rater reliability.

Overall, both reliability and validity testing were conducted only for five PATs; 13 had either reliability or validity testing conducted, while two had neither conducted. Reliability assessments were largely limited to Cronbach’s alpha, Cohen’s kappa, and inter-rater reliability, with test–retest reliability performed for only two of the 20 PATs. In terms of validity, content validity, face validity, criterion validity, and construct validity were most applied, while predictive and discriminant validity were rarely used.

Figure 5 summarizes the year-wise development of various PATs in relation to the framework’s scale development and validation criteria. While progress is evident, the heatmap illustrates the inconsistent sequential adherence to the criteria. It also highlights considerable and persistent gaps in validation that have remained unaddressed over the years.

### 3.3 Evaluation of the studies that employed the 20 unique PATs using study quality indicators

We standardized the evaluation of the studies that used the 20 unique PATs with respect to their sample size, type of study design and the five influencing factors (Age, Location, Season, Ethnicity and Family history) of Prakriti. This led to a comparative ranking of the Prakriti assessment tools (see Table 2):

**Table 2:** Comparative ranking of PATs using “Study Quality Indicators.”

No.	Tool name	Year of development	Framework score	Study sample size	Type of study design	Vaya (No. of age range sections)	Desha (No. of locations)	Jati (ethnicity)	Kula (family history)	Kala (season)
1	CCRAS-PAS software	2017	7	500	Observational cross-sectional	3.5	7	No info	No info	No info
2	ACPI scale- Ayurveda Child Personality Inventory	2014	7	230	Observational cross-sectional	0.5	1	No info	No info	No info
3	TSSC SAQ- Tridosha State Scale for Children	2013	6	108	Observational cross-sectional	0.3	1	No info	No info	No info
4	SAQ (126)	2023	4	210	Observational cross-sectional	1.3	1	No info	No info	No info
5	SAQ (116)	2021	3	250	Observational cross-sectional	0.8	1	No info	No info	No info
6	Mysore Tridosha scale (Software)	2011	3	1,548	Observational cross-sectional	2.6	1	No info	No info	No info
7	Mathew IAS rating Scale	2019	2	293	Observational cross-sectional	2.5	1	No info	No info	No info
8	Prakriti Assessment Tool (PAT)	2023	2	0*	Observational cross-sectional	0*	0*	No info	No info	No info
9	ML & Q (113)	2020	2	405	Observational cross-sectional	3.3	1	No info	No info	No info
10	SAQ Prakruti Dosha Mind Body Quiz and Vikruti Subdosha Questionnaire	2019	2	101	Exploratory cross-sectional	4.2	2	No info	No info	No info
11	SRQ (QDAV-R)	2015	2	173	Comparative study	3.3	1	No info	No info	No info
12	Sushruta Prakriti Inventory (SPI-Q & SPI-C)	2014	2	120	Observational cross-sectional	4.9	1	No info	No info	No info
13	ML & Q (99)	2016	1	67	Observational cross-sectional	**	1	No info	No info	No info
14	AyuSoft software (38, 135)	2010	1	112	Observational cross-sectional	2	3	No info	No info	No info
15	CPA & Q (118)	2022	1	50	Experimental study	**	1	No info	No info	No info
16	Prototype Prakriti Analysis Tool (PPAT)	2012	1	26	Observational cross-sectional	0.7	1	No info	No info	No info
17	SAQ (51)	2022	1	76	Observational cross-sectional	4.9	1	No info	No info	No info
18	SAQ (109)	2019	1	428	Observational cross-sectional	1.7	1	No info	No info	No info
19	SAQ-ABC	2013	1	20	Observational cross-sectional	0.2	1	No info	No info	No info
20	SRQ (102)	2017	1	19	Observational cross-sectional	3.4	1	No info	No info	No info

*The study only involved Ayurvedic Physicians for rating the items to be included in the scale and did not involve any subjects.

**The age range of the subjects was not reported in the study.

None of the 20 PATs considered or factored all five influencing factors of Prakriti- ethnicity (Jati), family lineage (Kula), age (Vaya), geographic location (Desha), and season (Kala).

Eighteen PATs were developed for use in adult population, whereas 2 PATs- the ACPI scale and TSSC-SAQ were developed for Prakriti assessment in children.

As for age range, both CCRAS-PAS software and Q (49) reported an age range of 18 or 20 to 60 years, respectively, which covers more than 3 sections of age, when the age range between 0 to 60 is distributed across 5 sections of 12 years each. While some other tools like SAQ Prakruti Dosha Mind Body Quiz and Vikruti Subdosha Questionnaire (28), Sushruta Prakriti Inventory (SPI-Q & SPI-C) (50), and SAQ (51) considered a wider age range, their sample size was found to be very less.

With regards to the type of study design employed for performing the validity and reliability testing of tools, 17 tools reported an observational cross-sectional study design, and 1 tool each reported an experimental design, exploratory study design and a comparative study design (see Table 3). Among the 20 PATs, no tool was developed using a longitudinal study design and only one tool, the Sushruta Prakriti Inventory (SPI-Q & SPI-C) was used in a prospective matched controlled trial. Six other PATs-Q (52), CCRAS Q, TNMC Prakriti 2004 Q, Q (53), Q (54), and CPA and CA of hand (52–57) have been used in randomized clinical trials (RCTs). But these tools were not previously validated, and two of these tools were validated within the RCT with a very limited study sample. The details related to this analysis can be found in Supplementary Appendix 2.

**Table 3:** List of PATs, their framework score and categories of measurable correlates to Prakriti studied using the tools.

Tool name	Framework score	Categories of measurable correlates
CCRAS Q^†^	–	Hematological, Genetic
CSIR software^†^	–	Genomic, Biochemical, Hematological, Genetic, Microbiome (Gut), Physiological
ML-Smart device data (129)^†^	–	Sleep Quality
Prakriti Q modified for T2DM (115)^†^	–	Biochemical, Genetic
PRAS-IPA software^†^	–	RGB scores
Q (107)^†^	–	Anthropometry, Biochemical
Q (23)^†^	–	Microbiome (Gut, oral and skin), Genomic
Q (103)^†^	–	Genetic
Q (89)^†^	–	Pharmacogenomic
Q (104)^†^	–	Karyotyping
Q (52)^†^	–	Hematological, Genetic
Q (108)^†^	–	Rate of disease incidence, Morbidity rate, Type of morbidity
Q (105)^†^	–	Anthropometry, Biochemical, Metabolomics
Q (110)^†^	–	Biochemical
RGB analysis (111)^†^	–	Hue Saturation Value space (HSV), RGB scores
SAQ (112)^†^	–	Anthropometry, Physiological, Neuropsychological tests
SAQ (120)^†^	–	Jatharagni
SAQ (117)^†^	–	Physiological
SAQ (93)^†^	–	Physiological, Hematological
SAQ (90)^†^	–	Stress Handling Capacity, Physiological, Anthropometry
SAQ (96)^†^	–	Skin type
TNMC Prakriti 2004 Q^†^	–	Physiological, Anthropometry, Biochemical, Microbiome (Gut and oral)
VIYETT Ayurvedic-Constitution Q^†^	–	Anthropometry
AyuSoft software*	–	Biochemical, Inflammatory markers, Lifestyle variables, Immunophenotyping, Genomic, Genetic, Epigenetic, Pharmacogenomic
	1*	Anthropometry, Place of birth
SAQ (109)	1	Anthropometry, Biochemical, Physiological, Genetic
SAQ Prakruti Dosha Mind Body Quiz and Vikruti Subdosha Questionnaire	2	Psychological states in terms of Vikruti
SRQ (QDAV-R)	2	Western personality constructs
CCRAS-PAS software	7	Pharmacodynamic, Cognitive and behavioral assessment scores, Genomic, Inflammatory markers

^†^Insufficient data related to tool validation.

^*^AyuSoft was used in 8 studies, primarily to explore objective correlates. Only one study conducted partial reliability testing; the rest lacked validation per the framework. Therefore, this tool has a score of both 0 and 1.

### 3.4 Investigating measurable correlates as biomarkers of Prakriti using different PATs

Here, we have identified 32 categories of measurable correlates that have been investigated in relation to Prakriti between 1987 and 2024. We have created a catalog of these 32 categories and the specific biomarkers that have been measured under each of these categories in Supplementary Appendix 3. In Table 3, we have listed the PATs which have been used in studying these measurable correlates to Prakriti. However, only 4 of the 32 categories of measurable correlates studied using the CCRAS-PAS software could be considered methodologically valid, owing to its high evaluation score. Tools like the CSIR software have been used to investigate extreme Prakriti types with genomic (33, 58–60), biochemical (33), physiological (61) and microbiome markers (24–26). Concurrently, other studies used tools such as AyuSoft (32, 62–66) to explore similar associations. But these tools were not previously validated. Also, all the identified measurable correlates have been studied independently and not integrated into the PATs. These findings highlight a critical gap in the current body of research on Prakriti.

Overall, the review found that of the 64 unique PATs identified, only 20 had undergone any form of validation or reliability testing. Among these, only two tools, the CCRAS-PAS software and the ACPI scale demonstrated notable methodological rigor as per the framework. While the CCRAS-PAS software was validated in adult populations, the ACPI scale was specifically developed and tested for use in children.

## 4 Discussion

**Figure 6:**
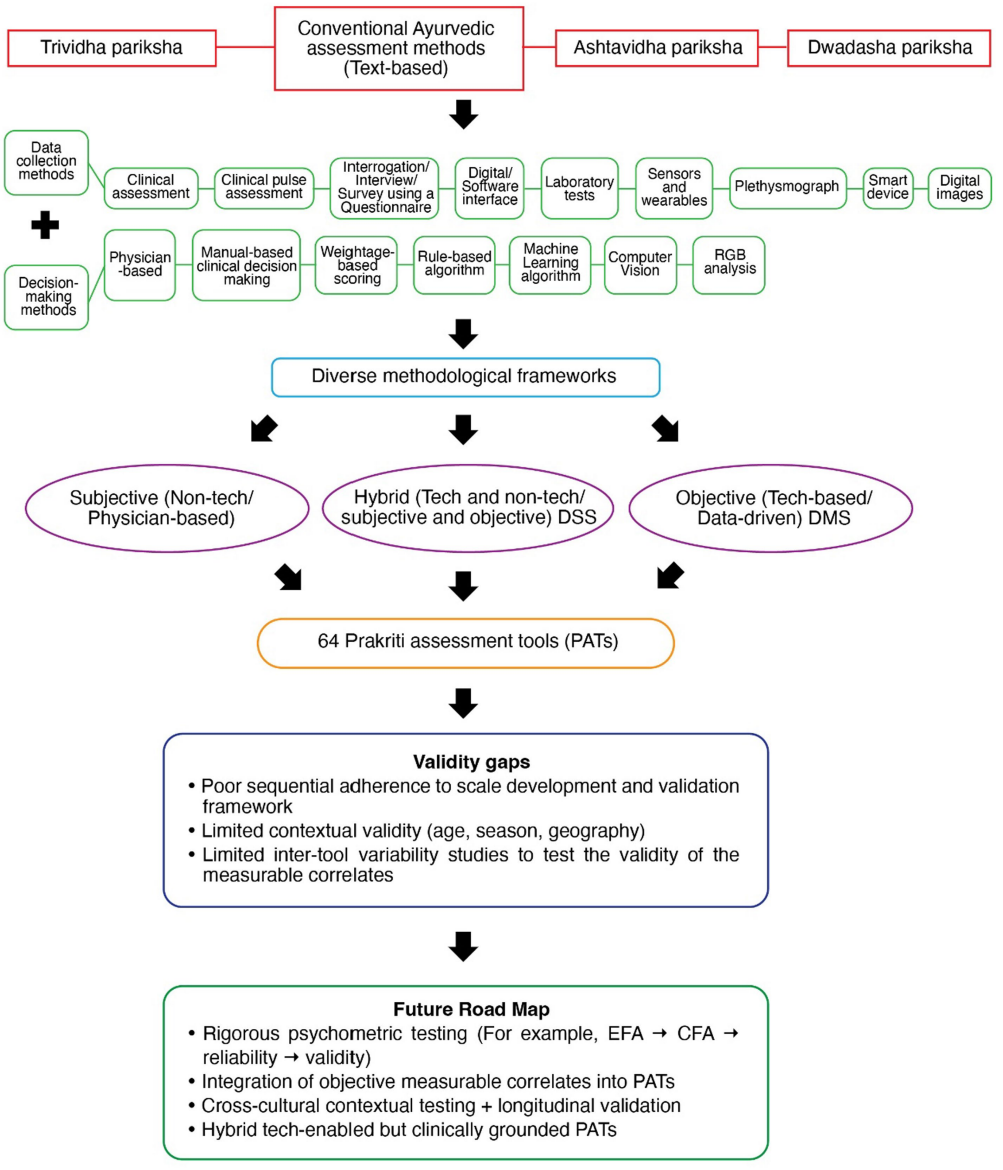
Mind map of Prakriti assessment: From conventional Ayurvedic assessments to development of PATs.

This review highlights an overall progression in Prakriti assessment from highly subjective, physician-dependent approaches toward more structured and data-driven decision-making systems. Early attempts to reduce subjectivity included consensus between multiple physicians, structured questionnaires with predefined scoring systems, followed by use of digital interfaces that standardized data entry and software applications along with standard operating procedures that introduced greater consistency in decision-making. Parallelly, sensors, wearables, and laboratory-based investigations have been used to explore measurable correlates of Prakriti, and pilot applications of Machine Learning and Computer Vision demonstrate enhanced objectivity and potential for scalability. However, our findings indicate that despite these advancements, most tools remain under-validated, with limited testing across diverse populations, thereby constraining their generalizability and scalability. Figure 6 illustrates a mind map of Prakriti assessment as per the overall findings of this review.

With specific reference to tool development, the identified PATs were generally developed based on textual descriptions that broadly outline only three types of Prakriti. These characteristics are not exhaustive and may not be sensitive enough to capture the full spectrum of Prakriti variations. On the other hand, the robustness of a PAT would depend upon a critical set of data heads and data points, that have been explored and factored from an exhaustive list. This exercise has not been performed, which is a critical gap. Technology-based assessments is an emerging area of research that enhances assessment methodology (67). Leveraging advanced technologies like generative Artificial Intelligence (AI) and ML offer the potential to identify latent dimensions of the Prakriti construct that are not explicitly described in classical Ayurvedic texts. This could pave the way for the development of more robust PATs.

As per the Validation and Evaluation framework, the phase I of tool development, requires the construct of the parameter being measured to be clearly defined within a theoretical or conceptual framework. The absence of such an approach has several implications. Apart from literature review, the conceptual and theoretical frameworks offer different dimensions on the topic of study that are essential to guide methodological decisions and elucidate critical insights (68). It is fundamental to scale development and improves the rigor and coherence of the scale (35). Secondly, the content validity of the scale is established at this stage. If the domains are not identified and clearly defined within a theoretical or conceptual framework, the items will not fully represent the construct’s domain, impacting the overall validity of the scale at the very beginning (69).

This phase also requires the generation of a comprehensive item pool using both deductive and inductive approaches. Importantly, item development must extend beyond the researchers’ subjective conceptualization to capture the full breadth of the target construct. As recommended by Kline (70) and Schinka et al. (71), the initial item pool should ideally be at least twice the length of the intended final scale.

Some researchers have attempted to provide a molecular framework for Prakriti with respect to stratified medicine (72), drug discovery and advancement for personalized care (73) and precision and integrative medicine (5). However, these frameworks are not suitable for a scale or tool development itself. There are two theories fundamental to any scale or tool development- the Classical Test Theory (CTT) and Item Response Theory (IRT) (74) that will ensure deriving functionally valid items specific to the construct of the domain of interest (35).

Under Phase II, critical steps like item reduction and extraction of factors that involve the use of standard methods such as item difficulty index, item discrimination test, inter-item and item-total correlations, distractor efficiency analysis and deleting or imputing missing cases were missing or not clearly explained. The item difficulty step is crucial to make sure only consistent items are included in the scale. Reynolds et al., has particularly highlighted the challenge of choosing the right procedures to ensure proper item-selection decisions that improves the tool’s overall validity (75). The type of responses like binary response or multiple-choice response or categorical items will also determine the choice of these procedures (40).

Under Phase III, dimensionality testing is crucial as it enables the accurate mapping of factors that specifically contribute to each construct, minimizing overlap and enhancing construct clarity. It is also very important to do the dimensionality testing at a different time point with the same sample or on a new sample (76). Performing dimensionality testing using statistical methods can bring about both conceptual clarity and empirical validation of tools measuring complex constructs (77).

Another major concern with respect to Dimensionality testing was the use of Confirmatory Factor Analysis (CFA) without first conducting Exploratory Factor Analysis (EFA), while developing the CCRAS-PAS software. While EFA is used to identify the underlying factor structure, CFA is used to test and confirm hypothesized models based on theory (78). Bypassing EFA step while performing sampling in phase II implies that the latent structure of the questionnaire was not adequately explored before confirming it which can lead to construct underrepresentation (79). This will also impact the subsequent steps of phase III- dimensionality, validity and reliability testing. Therefore, though the CFA under Dimensionality testing has been performed for CCRAS-PAS software, it has been done in the absence of EFA, suggesting that the dimensionality testing may not accurately represent the underlying construct.

Overall, the absence of a structured and sequential approach, beginning with identifying data heads and data points within a theoretical framework, followed by exhaustive item generation, rigorous item reduction to derive a critical set of items, and subsequently progressing through, EFA, CFA, dimensionality testing, and other validation steps has not yet been systematically undertaken. Implementing these steps would be essential to ensure that the reliability and validity testing of PATs is carried out rigorously. Kyriazos et al. (80) emphasize that the scale development and standardization process must be both sequential and iterative to ensure greater reliability and validity. Morgado et al. (81) identified and highlighted limitations in the scale development process which when overlooked or not understood adequately limit the future applicability of the scale and also hinder its generalizability. Although many PATs have reported validity and/or reliability testing, these were often conducted without the preceding foundational steps, which limits the robustness and interpretability of their findings. It is also important to note that while reliability testing is necessary, it is not sufficient on its own as it is a sample-dependent and context-dependent process (82). On the other hand, validity testing is a continuous process that needs to be performed throughout the scale development and evaluation process. For example, Boateng et al. (35) recommend validation across all three phases: content validity during item development (Phase 1), internal structure testing using EFA and CFA during scale development (Phase 2), and criterion and construct validity during scale evaluation (Phase 3).

In addition to tool evaluation, we also evaluated the studies that employed these tools. We incorporated study quality indicators, based on sample size adequacy which is very crucial and has a significant impact on research outcomes (83). With regards to sample size- three tools stood out. The CCRAS-PAS software considered a sample size of 500, but it has taken these samples from 7 different locations, limiting the number of study samples per location. Including 7 locations could have proven beneficial if the influence of location was factored in along with a larger sample size. Another ML-based tool, ML & Q (49) reported a higher overall sample size (n = 405), however the testing sample was only 81. This may be too low for the multiclass classification to get statistically significant values of precision and recall. Also, there is limited information on demographic diversity that would affect the model generalizability, when applying to diverse populations. A recommended testing sample is around 20–30 samples per category or domain (84). In this case, there are 7 categories, therefore about 140–210 samples in the test set would be required. Though the Mysore Tridosha Scale (39) was tested on the largest sample size (*n* = 1,548), it was limited in scope due to its primary focus on psychological expressions of doshas, and did not look at the other domains like physical features, behavioral traits etc.

With respect to the types of study designs, most of the tools (*n* = 17) were developed using an observational cross-sectional study design which is acceptable but none of these tools were additionally tested using controlled studies, implying lack of clinical validation. Clinical trials are fundamental to evidence-based practice that validates and informs clinically relevant research, requiring periodic testing and updating (85). The paucity of controlled studies poses a major constraint on the predictive capacity and clinical relevance of the PATs. To translate Prakriti into a measurable parameter in personalized medical care, systematic tool development and validation must occur before and alongside their use in trials.

Collectively, the study quality indicators set the tone for establishing contextual validity and wider applicability (across different cultures, ecology, language etc.) of the PATs. At present, even the PATs with the highest Framework scores have not accounted for key influencing factors of Prakriti, thereby limiting their generalizability. But even before pursuing multiple contexts and scalability, a PAT must first establish validity and reliability within at least one clearly defined context. This requires a methodical approach- from establishing a conceptual framework, generating context-sensitive items, scale development, and evaluation ensuring both relevance and repeatability. Frongillo et al. describe validity and “cross-context equivalence” of measures and discuss the methods to establish them (86).

While the scientific validation of Prakriti assessment is still a work under progress, studies continue to explore associations of Prakriti with genomic, biochemical, microbiome and other markers using different PATs. Over the past two decades, significant strides have been made to identify objective measurable correlates as biomarkers of Prakriti, especially by the Ayurgenomics study initiated by CSIR-TRISUTRA consortium (87). The heterogeneity of findings across these studies hinders the identification of a definitive biomarker(s) for Prakriti classification.

Multiple PATs have been involved in studying the measurable correlates to Prakriti, but no replication of studies have been performed using same correlates and different PATs. Such inter-tool variability studies would help establish the methodological robustness of the PATs and also the validity of these measurable correlates.

Though, currently there are no established biomarker(s) for Prakriti classification, there are some domains more prominently studied than the others such as Biochemical, Anthropometry, Genomic, Physiological, Genetic, Microbiome, Inflammatory markers and Hematological parameters. We propose a framework for prioritizing the existing domains. We could look at four criteria: (i) Replicability: Same biomarkers must be studied in multiple, independent cohorts, using different PATs. Currently, only biochemical parameters like lipid profile and blood glucose and anthropometric parameters like height, weight, BMI alone have been replicated in multiple independent cohorts, which have not shown any promising links to the tridoshas. Other domians need to be replicated too; (ii) Biological plausibility: links to dosha theory [e.g., PGM1 gene with Pitta phenotype (32)]; however most of the studies that explored measurable correlates to prakriti have only considered extreme constitution types, limiting its applicability to all constitution types, as a majority of population would belong to dual constitution types; (iii) Integration capacity: ability to combine with digital PATs (e.g., genomic or metabolomic data linked to questionnaires/software); (iv) Clinical translational potential: feasibility of testing in real-world settings (e.g., microbiome profiling is becoming cheaper and more accessible).

Based on the current evidence, Prakriti seems to represent a polygenic, systems-level phenotype. Therefore, rather than a single biomarker, a multi-omic, integrative approach combining genetic, microbiome, immunological and metabolomic parameters alongside validated PATs appears most promising. While it may not be feasible to have too many tests as a part of Prakriti assessment, future studies should prioritize biomarkers that are replicable across populations, clinically feasible, and theoretically aligned with Ayurvedic concepts of Prakriti.

## 5 Limitations

This study has certain limitations, including the exclusion of non-English language journals and Indian databases. Secondly, as this was not a systematic review, the methodology was not pre-registered in a review registry such as PROSPERO before initiating the review. Finally, in the absence of a dual review, we may not have eliminated bias completely.

## 6 Conclusion

Despite the proliferation of numerous Prakriti assessment tools, inadequate adherence to standardized protocols for development, validation and reliability testing leaves major gaps in methodological rigor and robustness of available tools. This hampers both the effective utilization of technology and the progression of Ayurvedic research. Moving forward, the adoption of structured approaches for tool development and validation, including rigorous item development, dimensionality, validation, and reliability testing is essential. This can then be followed by cross-cultural validation studies and non-developer testing through multi-center and cross-context trials (India and diaspora populations). Furthermore, integrating advanced technologies and incorporating measurable correlates within the Prakriti assessment tools will make it more robust, clinically relevant and suitable for integration into mainstream personalized health care.
